# On Certain Pathological Conditions of the Milk as the Cause of Disease in Infants

**Published:** 1846-04

**Authors:** 


4. *On certain Pathological conditions of Milk as the cause of disease in Infants.*—*M. Albert Donné*, in his *Cours de Microscopie*, (Paris, 1844, page 412,) observes, "Our ignorance in the present day with regard to the characters of good and bad milk in nurses, and the mode of distinguishing that which possesses qualities requisite for the life and health of the child, from that which only affords to it an unwholesome kind of food, is so great, that it is almost impossible to find a practitioner, nurse, or even chemist, capable of giving an opinion whether a given specimen of milk be of good or bad quality. The indifference with which this important question is regarded, is no doubt in great measure attributable to the difficulty of the subject, to the insufficiency of the results which chemical analyses have hitherto afforded, and to the want of a proper method in the examination of this substance. We cannot in reality attribute it to any lack of interest, or to the trifling importance of the question, for there is perhaps none which in a higher degree concerns the public health, the happiness and welfare of families, or which more frequently presents itself for solution; and I have no hesitation in stating, that all which has hitherto been said and written on the subject of milk, so far at least as regards its peculiar qualities in relation to the nourishment of infants, is absolutely valueless. No one, certainly, is likely to be deceived by the colour, consistence, or even taste of milk; yet nothing can be more vague than are such characters; it is impossible to attach any real value to them; and since they are based on nothing positive, each person may interpret them as he pleases; consequently the attention of medical men is directed much rather to the general health of nurses than to the properties of their milk; and the examination of this secretion, if undertaken, is performed merely as a matter of form. Undoubtedly the general health is an indispensable condition, and one to which especial attention ought to be directed in the selection of a nurse; yet this condition is far from being the only one deserving of consideration, and it is well known that the best health is not always a guarantee for the good qualities of a nurse, or the nutritive properties of her milk; the lacteal secretion may be insufficient, or abnormal, in a woman otherwise perfectly healthy. Is it not a matter of daily observation, that one woman, although of a meagre sickly appearance, makes a better nurse than another woman of the healthiest aspect; and are we not frequently deceived as to the state of the constitution by external appearances? It is evident that the organs endowed with the function of secreting milk are, so to speak, placed too much without the general economy, to allow of the qualities of this secretion being estimated by the integrity of other organs and the regularity of other functions. It is in the milk itself, therefore, that we must search for the characters of its good and bad qualities; and until we possess the means of observing its properties, and its good or bad nature in relation to the nourishment of infants, practice will be deprived of rule, the choice of nurses will be made in an empirical manner, and the determination of mothers who wish to suckle will more frequently be regulated by chance or caprice, than by reason, or with a due regard to the interest of their children." The subject has recently attracted the attention of M. Girard, who has furnished the following cases and observations. (*Archives Générales de Médecine*, June, 1845.)

Case I.—In September, 1840, a child, aged five months, was brought to me. I was informed that it was strong and vigorous when born, and that it was at once delivered to the charge of a nurse, who had been suckling for fourteen months. It shortly became uneasy, cried incessantly, and was only quiet when at the breast; it gradually grew thin, and diarrhœa was established, the stools being of a green colour. When brought to me it presented the following condition: Its face was thin and pale, tongue red, with a few scattered aphthous points; belly tense; there was a bright erythematous redness over the thighs and nates; there was frequent diarrhæa, the stools green; vomiting of curdled milk several times a day; the child slept badly, frequently awaking. This was the third time the child had been attacked with an almost similar set of symptoms, except that the aphthous spots now appeared for the first time, and the attack generally was more severe than the former ones, which had disappeared under the use of baths, starch injections, and abstinence from food; the diarrhœa, however, had continued. Baths, injections, gargles were now in vain made use of; the diarrhœa obstinately remained, and the aphthous spots increased. The nurse's milk was very alkaline; it was not examined microscopically. Since the child did not mend, it was determined to change its milk, and a nurse was engaged who had only been suckling for three months. The beneficial effects of this change were very marked; in two days the diarrhœa had considerably abated, and after a week all the symptoms finally disappeared.

Case II.—Madame S., aged 25, was on the 14th of November, 1844, delivered of her first child, a male, strong and well formed; she suckled this child for ten days, at which time her breasts becoming enlarged and painful, a nurse was engaged. This nurse was a middle-sized, dark-complexioned woman, about 30 years of age. She had no appearance of disease; her breasts were small; her milk was sweet, of good colour, consistence, and quantity, and about three weeks old. The child when delivered to her charge was in good condition, and its evacuations were healthy; but in a few days its sleep became disturbed; it grew thin; its stools became liquid, and very frequent, sometimes green, at others black; it had nausea and vomiting; a bright redness extended over the thighs and nates, and the child became very restless. On the 3d of December it presented the follow appearances: emaciation extreme; skin dry and rough; diarrhœa frequent; stools green; the belly tense and painful; extensive erythema over the surface of the body; some vesicles on the scrotum; constant vomiting after taking the least quantity of liquid or milk; tongue red, and, as well as the mucous membrane lining the lips and cheeks, covered with numerous aphthous spots. I prescribed bran baths, water containing white of egg for drink, injections of linseed infusion with a drop of laudanum twice daily, and poultices to the abdomen. In spite of this treatment, however, the symptoms became more intense, the erythema extended, the aphthous spots became more confluent, ecthymatous pustules formed on the legs, the diarrhœa became more frequent, and the emaciation increased. This state of things continued until the 9th of December, when the nurse's milk was examined microscopically by M. Duforsé, and the following results obtained:—There was nothing peculiar in its colour; its consistence was that of milk containing much cream; treated with ammonia it became slightly viscous; it was neither acid nor alkaline. When a drop of this milk was examined with a microscope magnifying 300 diameters, it was observed, 1st, that the milk globules were in great abundance, such as is found to be the case in very rich milk; they were generally of a considerable size, and the largest resembled small bladders half filled with liquid, and collapsed. Instead of having a pearl-like brilliancy, most of them, especially the large ones, were of a dull white colour, somewhat resembling opal; some of them, aggregated together, formed small groups, which could be moved about in all directions, without a single globule being detached. When submitted to slight pressure, these several groups spread out so as to occupy a surface five or six times greater than they presented at first, and they assume various forms. The smallest quantity of sulphuric ether introduced between the plate of glass dissolved a large quantity of them very rapidly. 2d. The field of the microscope was beset with roundish granular particles, perfectly colourless, and presenting all the characters described by J. Henlé, Donné, Mandl, Güterbrok, and other micographers.

[To these particles Donné first applied the name of *corps granuleux*, and describes them as invariably existing in colostrum, but disappearing gradually as the milk becomes older; so that after about the twentieth day, and usually much sooner, not a trace of them is to be found. They differ from ordinary milk globules (with which they co-exist) in form, size, general aspect, and internal composition. They are not always globular, but present all possible varieties of form, and also of size, the smallest being about one-hundreth of a millimetre, the largest many times this size; they are slightly transparent, usually of a yellowish colour, and of a granular aspect, appearing as if composed of a number of small granules aggregated together, or enclosed within a transparent envelop. Very often there exists in the centre or some other point of these little heaps a single globule, which is apparently nothing but a true milk globule imprisoned within the granular matter. The nature of these granular bodies is unknown; Donné supposes that they consist of fatty matter, and a peculiar mucous substance; they are not soluble in alkalies, but like true milk globules dissolve in ether, and after the evaporation of this reagent small heaps of acicular crystals remain on the glass. (*Cours de Microscopie*, par Alb. Donné, p. 400.) Although the existence of these granular bodies is commonly peculiar to colostrum alone, yet Donné (page 421), observes that they and the other peculiarities of the colostrum (as the large irregular size of the milk globules, which, instead of floating free, are agglomerated together in small masses), may persist for many months, or even to the end of suckling. The existence of this condition can only be discovered by the microscope, for the ordinary physical properties of milk, such as whiteness, consistence, and other characters, are preserved; and the nurse may continue in perfect health: the child, however, usually grows thin, although it is continually at the breast, and it commonly becomes attacked with diarrhœa. The milk in this case of M. Girard seems to have retained many of the characters peculiar to colostrum; he thus continues the narration of it:—]

The propriety of changing the nurse was now suggested and adopted: the milk of several was examined microscopically, and one selected whose milk appeared perfectly pure. This change had scarcely been effected two days, when the diarrhœa and vomiting diminished, and speedily ceased altogether; the aphthous spots disappeared, the tongue resumed its natural colour, and the erythema faded. From this time the child speedily recovered its good looks, and became fat, its stools being natural, and sleep good.

Case III.—Madame R., aged 28, was delivered of her seventh child in February, 1842; a male, strong, and well formed. One of her children had died when six months old from an affection characterized by ardent thirst, extreme emaciation diarrhœa, with green stools, and glairy vomiting. The present child took the breast readily, and was apparently in good health, yet vomited occasionally after suckling; the milk to all appearance was perfectly good. About the beginning of the second month the vomitings increased in frequency. Supposing that the child filled its stomach too full, the breast was given to it less frequently, and a little *eau sucrée* substituted, yet after each time of taking the breast it still vomited, though it could retain other liquids; it soon grew thin and pale, and its bowels were alternately constipated and relaxed.

Towards the middle of the second month, the following symptoms suddenly occurred; the child screamed out, ceased to breathe, and became unconscious, its face and hands assuming a livid hue: this condition lasted for a few seconds, and then passed off spontaneously, leaving the child weak and faint for some hours. Within the next twenty days the child had many similar attacks, which came on at uncertain periods, both day and night, without any obvious cause; blisters, antispasmodics, and baths were employed, but without benefit. The vomiting still continued. The milk was now examined microscopically several times, at intervals of some days, and was found to present an enormous quantity of mucus without any other alteration. I informed the mother that it was essential the child should have other milk; this was repugnant to her, and she requested a few days' delay. Eight days afterwards, the vomitings having diminished, the milk was again examined, and presented a dimunition in the quantity of mucus; but it again increased after a few days, and with this increase the vomitings returned as before. The child continued to grow thin; a little diarrhœa showed itself, and the chest affection remained. The mother, now becoming alarmed, consented to employ a nurse: the milk of seven different women who successively offered themselves, although to all appearance good, presented beneath the microscope either mucus granular bodies, or other alterations; therefore they were rejected. At length one was obtained whose milk microscopically was perfectly pure. Two days after taking this milk the vomitings entirely ceased, so also did the symptoms of asthma, and neither of them ever reappeared; the child speedily became fat, strong, and well, and remains so to the present time.

At the conclusion of these cases, M. Girard remarks, that "without wishing to generalize too much, or to establish a theory from a few facts, is it not, however, logical to observe here a relation of cause and effect? What do we see in the second case? A severe and frequently fatal affection, which was rapidly on the increase, had resisted all rational means adopted for its removal, and which yielded with the greatest facility to a change in the milk with which the child was fed. We observe this disease to coincide with the ingestion of milk impure and of bad quality, and we witness its disappearance with a truly marvelous rapidity as soon as milk of good quality is administered. And in the third case, although the symptoms were somewhat different, yet we observe them to occur coincidently with the ingestion of impure milk, and to cease when milk of a pure quality is substituted. Is it unreasonable to conclude that certain severe pathological conditions may be produced by alterations in the milk alone, and may be dissipated even when they have attained a very high degree, by a return to milk of good quality? It would be a point of much importance to ascertain whether these alterations in the condition of milk could at any time coincide with the maintenance of perfect health in the child; also it would be important to determine, if possible, whether a given alteration in milk most commonly or constantly induces such or such a pathological affection. Thus of the two cases last narrated we observe that in one a granular state of the milk induced an aphthous affection (the muguet), whilst in the other, a mucous condition gave rise to symptoms referable to the stomach and to the lungs; at any rate that these states were coincident with such affections. Of course it is not meant to be here implied that the pathology of infants is entirely under the influence of milk, but it seems probable that many hitherto inexplicable conditions may be so, and, moreover, that they might be explained by a simple examination of this liquid."—*Ibid.*

